# Self-reported peripheral neuropathic symptoms in long-term adolescent and young adult (AYA) cancer survivors: results of the SURVAYA study

**DOI:** 10.1007/s00520-026-10912-7

**Published:** 2026-07-02

**Authors:** Danique A. Scheltes, Silvie H. M. Janssen, Rhodé M. Bijlsma, Suzanne E. J. Kaal, Jan Martijn Kerst, Jacqueline M. Tromp, Monique E. M. M. Bos, Tom van der Hulle, Roy I. Lalisang, Janine Nuver, Mathilde C. M. Kouwenhoven, Winette T. A. van der Graaf, Floortje Mols, Olga Husson

**Affiliations:** 1 Department of Medical Oncology, Netherlands Cancer Institute – Antoni van Leeuwenhoek, 1066 CX Amsterdam, The Netherlands; 2 Clinical Sciences for Health Professionals, Program in Clinical Health Sciences, University Medical Center Utrecht, Utrecht University, 3584 CX Utrecht, The Netherlands; 3 Radiotherapiegroep, 6815 AD Arnhem, The Netherlands; 4 Department of Medical Oncology, University Medical Center, 3584 CX Utrecht, The Netherlands; 5 Department of Medical Oncology, Radboud University Medical Center, 6525 GA Nijmegen, The Netherlands; 6 Department of Medical Oncology, Amsterdam University Medical Centers, 1105 AZ Amsterdam, The Netherlands; 7 Department of Medical Oncology, Erasmus MC Cancer Institute, Erasmus University Medical Center, 3015 GD Rotterdam, The Netherlands; 8 Department of Medical Oncology, Leiden University Medical Center, 2333 ZA Leiden, The Netherlands; 9 Division of Medical Oncology, Department of Internal Medicine, Maastricht, UMC+ Comprehensive Cancer Center, GROW-School of Oncology and Reproduction, Maastricht University Medical Center+, 6229 HX Maastricht, The Netherlands; 10 Department of Medical Oncology, University Medical Center Groningen, 9713 GZ Groningen, The Netherlands; 11 Department of Neurology, UMC, Cancer Center Amsterdam, Amsterdam University Medical Centers, Location VUmc, Amsterdam, 1081 HV The Netherlands; 12 Department of Medical and Clinical Psychology, Tilburg University, 5000 LE Tilburg, The Netherlands; 13 Research and Development, Netherlands Comprehensive Cancer Organization, 3511 DT Utrecht, The Netherlands; 14 Department of Surgical Oncology, Erasmus MC Cancer Institute, Erasmus University Medical Center, 3015 GD Rotterdam, The Netherlands; 15 Department of Public Health, Erasmus MC Cancer Institute, Erasmus University Medical Center, 3015 GD Rotterdam, The Netherlands

**Keywords:** Adolescent and young adults, Cancer, Survivorship, Peripheral neuropathic symptoms

## Abstract

**Purpose:**

Adolescent and young adult cancer survivors (AYAs; 18–39 years old at initial cancer diagnosis) face unique challenges throughout their disease trajectory and are, therefore, a distinct population within the oncology community. A common side effect of some cancer treatments is neuropathy, which can affect patients’ quality of life ongoing. AYA-specific studies on long-term effects, such as neuropathy, are lacking. This study investigated the prevalence of, and factors associated with self-reported peripheral neuropathic symptoms in long-term AYA cancer survivors.

**Methods:**

This questionnaire study (SURVAYA study) examined patient-reported outcomes among long-term AYA cancer survivors (5–20 years post-diagnosis). Data were collected through a questionnaire and by the Netherlands Cancer Registry. Analyses included descriptive statistics and multivariable logistic regression.

**Results:**

Three thousand seven hundred forty-one AYAs were included in this secondary analysis. Overall, the prevalence of self-reported peripheral neuropathic symptoms was 19.8% and was higher among AYA cancer survivors who received chemotherapy. In addition, female sex, older age at diagnosis, primary education or none, secondary vocational education, higher vocational education, head and neck cancer, germ cell tumours, lymphoid haematological malignancies, thyroid cancer, current or former smoking, higher physical activity levels and rheumatoid arthritis were associated with a higher likelihood of reporting peripheral neuropathic symptoms.

**Conclusion:**

This study highlights the socio-demographic, clinical and health and lifestyle factors associated with self-reported peripheral neuropathic symptoms in Dutch long-term AYA cancer survivors. This can inform healthcare providers to further identify high-risk groups within the AYA cancer survivors and provide additional monitoring of these patients. Future research should include a longitudinal assessment of peripheral neuropathic symptoms, including baseline scores which were not available in this study.

**Supplementary Information:**

The online version contains supplementary material available at 10.1007/s00520-026-10912-7.

## Introduction

While cancer predominantly impacts older adults, more than 1.2 million adolescent and young adults (AYAs) aged 15–39 years receive a primary cancer diagnosis each year [1]. AYAs are recognised as a distinct population within the oncological spectrum, due to clinical differences in the type of cancer and the disease’s biology, when compared to paediatric (< 15 years) and older adult (> 40 years) cancer patients [2–6]. In addition, AYAs are at a unique stage of life, where age-appropriate milestones are achieved, such as completing education, starting a career, forming romantic relationships and starting a family [6, 7]. A cancer diagnosis and the subsequent treatment, can result in the emergence of short- and long-term side effects. Such side effects may affect the achievement of age-appropriate milestones and can reduce health-related quality of life (HR-QoL) [8]. The estimated 5-year survival rate for all AYAs with cancer combined is over 80%, resulting in many AYAs potentially living with long-term side effects [9, 10]. Therefore, attaining and preserving optimal HRQoL is an important aspect for AYAs [8].

Neuropathy is a side effect of cancer treatments that has a significant negative impact on the HRQoL [11]. Peripheral neuropathy is a change in sensation in the hands and feet at the end of the nerves due to damage to the peripheral nerves, resulting in pain, numbness, tingling and sometimes weakness [12]. Neuropathic symptoms can restrict patients’ body functions, making even simple daily tasks challenging, such as opening jars, buttoning up a blouse and impairing balance. This can lead to feelings of frustration and loss of self-confidence [11]. Currently, there is no effective treatment for neuropathic symptoms [13].

In the general population the prevalence of neuropathy is about 2.4% and increases to 8% in the elderly [14]. Studies on the prevalence of neuropathy in cancer patients have mainly focused on chemotherapy-induced peripheral neuropathy (CIPN). A recent systematic review among adult cancer patients reported a prevalence of CIPN of 30% in the long-term [15]. Moreover, studies among adult cancer patients show that the prevalence of neuropathy may be influenced by several socio-demographic, clinical and health and lifestyle factors other than chemotherapy, such as alcohol use, physical activity and diabetes [16, 17].

Despite many AYAs potentially living with late effects, limited research has been done on the incidence of and factors associated with peripheral neuropathic symptoms, in this group. Due to the differences with adult cancer patients in the type of cancer and the disease’s biology, the current knowledge on peripheral neuropathic symptoms cannot be generalised to the AYA population. To better tailor treatment and survivorship care guidelines to the AYA population, research is needed on the long-term and late effects of diagnosis and treatment impacting HRQoL, such as peripheral neuropathic symptoms. To achieve this, it is crucial to first determine the prevalence of peripheral neuropathic symptoms in the AYA population, as well as the associated socio-demographic, clinical and health and lifestyle factors. Therefore, the aim of this study is to determine the prevalence of self-reported peripheral neuropathic symptoms, and associated socio-demographic, clinical and health and lifestyle factors with these peripheral neuropathic symptoms in long-term AYA cancer survivors in the Netherlands.

## Methods

### Study design and data collection

This paper provides a secondary analysis of the SURVAYA (Health-related quality of life and late effects among SURVivors of Cancer in Adolescence and Young Adulthood) study. The primary objective of this observational, population-based, cross-sectional cohort study was to examine the prevalence, risk factors and mechanisms of impaired health outcomes among AYA cancer survivors in the Netherlands. The methods used in this study have previously been described in greater detail [18]. In short, the SURVAYA study included AYA cancer survivors diagnosed between 1999 and 2015, at the ages 18–39 years, in the one of the nine participating hospitals in the Netherlands and who were alive at study entry in May 2019. Data collection was conducted between May 2019 and June 2021 using the PROFILES registry (Patient Reported Outcomes Following Initial Treatment and Long-term Evaluation of Survivorship) [19]. All possible participants were identified by the Netherlands Cancer Registry (NCR), and they received an invitation for participation in the SURVAYA study from their (former) treating physician. Participants could complete the questionnaire online or on paper, after signing the informed consent form [18]. The dataset was completed by linking clinical data from the NCR with responses from the SURVAYA questionnaire. The study was approved by the Netherlands Cancer Institute Institutional Review Board (NCI IRB-IRBd18122) on February 6th 2019.

### Outcome measures

Self-reported peripheral neuropathic symptoms were assessed with a subscale of the European Organisation for Research and Treatment of Cancer Quality of Life Survivorship questionnaire (EORTC QLQ-SURV100) [20]. It was measured with two items “Have you experienced tingling or numb fingers or hands?” and “Have you experienced tingling or numb toes or feet?”. Both items were scored on a four-point Likert scale (1 = not at all; 4 = very much). For each AYA survivor, a raw neuropathic symptom score was calculated by adding up the item scores and dividing by the number of items (2 items), after which this raw score was transformed into a continuous score of 0–100 in accordance with the EORTC scoring guidelines [21]. This continuous score was subsequently dichotomized in order to facilitate the clinical interpretation of the results. Following previous recommendations for interpreting EORTC scores, participants with a score ≥ 10 points above the mean neuropathic symptom score were classified as having peripheral neuropathic symptoms [22]. This threshold was chosen to reflect a meaningful deviation from the average symptom level, consistent with prior work using EORTC scales. Throughout this manuscript, self-reported peripheral neuropathic symptoms refer to patient-experienced tingling and numbness. These items do not constitute a clinical diagnosis of neuropathy nor other forms of neuropathy.

Based on literature [16, 23] and clinical practices, in total, seventeen factors potentially associated with peripheral neuropathic symptoms were used in this study, including six socio-demographic, four clinical and seven health- and lifestyle factors. Regarding the socio-demographic variables, age at diagnosis, sex and social economic status (SES) were available from the NCR. SES scores are derived from the standardised income per household, derived from the Dutch postal code [18]. Educational level, partner status and living situation were self-reported by respondents in the SURVAYA-study questionnaire.

The four clinical factors, tumour type, tumour stage, primary treatment and time since diagnosis, were all available from the NCR. Tumour type was classified according to the third International Classification of Disease for Oncology (ICDO-3) [24]. Tumour stage was classified according to the Tumour Node Metastases (TNM) or Ann Arbor Code [25].

The seven health and lifestyle factors in this study, body mass index (BMI), physical activity (PA), diabetes mellitus, rheumatoid arthritis, alcohol consumption, smoking status and substance use were all self-reported by respondents in the SURVAYA-study questionnaire. BMI was calculated with the self-reported body height and weight. PA was assessed with items derived from the validated European Prospective Investigation into Cancer (EPIC) Physical Activity Questionnaire [26] and was represented as hours/week of moderate and vigorous physical activity (MVPA). To calculate MVPA, metabolic equivalent intensity (MET) values were assigned to each activity, according to the Compendium of Physical Activities [27, 28]. Activities were then identified as MVPA (MET ≥ 3). Total PA was calculated by summing the hours/week of all MVPA activities (walking, cycling, gardening and sports). Diabetes mellitus and rheumatoid arthritis were assessed using a short version of the questionnaire used in the St. Jude Childhood Cancer Survivorship study [29].

### Statistical analyses

According to the EORTC guidelines [21], if at least half of the items from an EORTC scale are answered, the score is calculated assuming the missing items have values equal to the average of the other items, which are present for that respondent. When > 50% of the questions are unanswered, the scale score is considered missing and the participant is excluded from further analyses. If one of the two items on the peripheral neuropathic symptoms scale was answered, the participant is included in the analysis. Outliers indicating measurement errors were marked as missing. The proportion of missing data in the dataset was minimal; therefore, it is unlikely that the imputation materially affected the results.

The study population was described with means (including standard deviations (SD)) and frequencies (with percentages), stratified by peripheral neuropathic symptoms (“peripheral neuropathic symptoms vs. “no peripheral neuropathic symptoms”). Chi-square and independent t-test were used to assess differences between the groups with and without peripheral neuropathic symptoms data.

The prevalence of peripheral neuropathic symptoms was determined with descriptive statistics and was reported as a percentage. To assess the association between potential factors and peripheral neuropathic symptoms multivariable logistic regression analyses were conducted by including the seventeen potentially associated factors, based on a priori clinical and theoretical relevance, independent of their univariable significance. The number of events per variable (EPV) remained sufficient to support the inclusion of these variables. The variable selection process for the multiple regression analyses uses the forced entry (enter) method and multicollinearity was assessed using the tolerance statistic < 0.1, the variance inflation factor > 10 and variance proportions ≥ 0.7. Odds ratios for each independent variable, along with the corresponding 95% confidence interval and *p*-value were provided. To ensure stability of the multivariable logistic regression model, the number of events per variable (EPV) was set according to the recommendation by Peduzzi et al. With seventeen independent variables included in the model, a minimum of 10 events per variable was required, resulting in at least 170 events [30]. In addition to the multiple logistic regression, a sensitivity analysis was performed using multiple linear regression (enter method) in which neuropathy was included as a continuous outcome measure. Data analyses were conducted using SPSS, version 29 and all statistical tests were two-sided with a *p*-value < 0.05 for statistical difference.

## Results

### Patient and tumour characteristics

A total of 4010 AYAs participated in the SURVAYA study, representing a response rate of 36%. In total, data on self-reported peripheral neuropathic symptoms were available for 3741 participants (Appendix 1). Differences in the population characteristics of AYAs with and without self-reported peripheral neuropathic symptom data are shown in Table A1 (Appendix 2). Only BMI was significantly different between both groups, with a higher BMI in the group of AYAs with available data on peripheral neuropathic symptoms (i.e. AYAs who are included in the following analyses).

A participant was classified as having self-reported peripheral neuropathic symptoms if the neuropathic symptoms score was ≥ 21.9. This resulted in a total of 739 (19.8%) AYA survivors experiencing self-reported peripheral neuropathic symptoms. Table 1 describes the characteristics of the AYA survivors, stratified on having self-reported peripheral neuropathic symptoms. The mean age of the AYA survivors at diagnosis was 32 (SD 5.9) years, and the mean time since diagnosis was 12.4 (SD 4.5) years. Most AYA survivors were female (61.1%) and diagnosed with stage I disease (42.8%).

**Table 1:** Socio-demographic and clinical characteristics of the AYA cancer survivors stratified based on self-reported peripheral neuropathic symptoms

	Total ***N*** = 3741	Peripheral neuropathic symptoms ***N*** = 739	No peripheral neuropathic symptoms ***N*** = 3002
	*N* (%)	*N* (%)	*N* (%)
Sex			
Male	1456 (38.9)	267 (36.1)	1189 (39.6)
Female	2285 (61.1)	472 (63.9)	1813 (60.4)
Age at diagnosis, in years, mean (SD)	31.6 (5.9)	32.2 (5.9)	31.4 (5.9)
Partner status			
Partner	3109 (83.1)	579 (78.3)	2530 (84.3)
Single	619 (16.5)	155 (21.0)	464 (15.5)
Missing	13 (0.3)	5 (0.7)	8 (0.3)
Living situation			
Together^a^	3282 (87.7)	104 (14.1)	355 (11.8)
Alone	459 (12.3)	635 (85.9)	2647 (88.2)
Socio-economic status			
Low	499 (13.3)	106 (14.3)	393 (13.1)
Intermediate	1151 (30.8)	235 (31.8)	916 (30.5)
High	2077 (55.5)	393 (53.2)	1684 (56.1)
Missing	17 (0.4)	5 (0.7)	9 (0.3)
Educational level			
Primary education, or none	24 (0.6)	11 (1.5)	13 (0.4)
Secondary education	250 (6.7)	54 (7.3)	196 (6.5)
Secondary vocational educational	1354 (36.2)	298 (40.3)	1056 (35.2)
Higher vocational education	1276 (34.1)	261 (35.3)	1015 (33.8)
University education	829 (22.2)	114 (15.4)	715 (23.8)
Missing	8 (0.2)	1 (0.1)	7 (0.2)
Tumour stage			
I	1600 (42.8)	278 (37.6)	1322 (44.0)
II	990 (26.5)	201 (27.2)	789 (26.3)
III	536 (14.3)	124 (16.8)	412 (13.7)
IV	172 (4.6)	37 (5.0)	135 (4.5)
Unknown	443 (11.8)	99 (13.4)	344 (11.5)
Type of cancer			
Bone and soft tissue sarcomas	165 (4.4)	29 (3.9)	136 (4.5)
Breast	885 (23.7)	173 (23.4)	712 (23.7)
Central nervous system	142 (3.8)	28 (3.8)	114 (3.8)
Colon and rectal	76 (2.0)	17 (2.3)	59 (2.0)
Digestive tract, other	30 (0.8)	6 (0.8)	24 (0.8)
Female genitalia	407 (10.9)	81 (11.0)	326 (10.9)
Germ cell tumours (all sexes)	652 (17.4)	126 (17.1)	526 (17.5)
Head and neck	116 (3.1)	22 (3.0)	94 (3.1)
Lymphoid haematological malignancies	555 (14.8)	125 (16.9)	430 (14.3)
Male genitalia	5 (0.1)	0 (0.0)	5 (0.2)
Melanoma	262 (7.0)	28 (3.8)	234 (7.8)
Myeloid haematological malignancies	140 (3.7)	34 (4.6)	106 (3.5)
Respiratory	27 (0.7)	7 (0.9)	20 (0.7)
Thyroid	230 (6.1)	57 (7.7)	173 (5.8)
Urinary tract	39 (1.0)	4 (0.5)	35 (1.2)
Other	10 (0.3)	2 (0.3)	8 (0.3)
Surgery^b^			
No	818 (21.9)	191 (25.8)	627 (20.9)
Yes	2919 (78.0)	545 (73.7)	2374 (79.1)
Missing	4 (0.1)	3 (0.4)	1 (0.0)
Chemotherapy^b^			
No	1633 (43.7)	255 (34.5)	1378 (45.9)
Yes	2104 (56.2)	481 (65.1)	1623 (54.1)
Missing	4 (0.1)	3 (0.4)	1 (0.0)
Radiotherapy^b^			
No	1959 (52.4)	371 (50.2)	1588 (52.9)
Yes	1778 (47.5)	365 (49.4)	1413 (47.1)
Missing	4 (0.1)	3 (0.4)	1 (0.0)
Hormonal therapy^b^			
No	3282 (87.7)	637 (86.2)	2645 (88.1)
Yes	455 (12.2)	99 (13.4)	356 (11.9)
Missing	4 (0.1)	3 (0.4)	1 (0.0)
Targeted therapy^b^			
No	3447 (92.1)	684 (92.6)	2763 (92.0)
Yes	290 (7.8)	52 (7.0)	238 (7.9)
Missing	4 (0.1)	3 (0.4)	1 (0.0)
Stem cell therapy^b^			
No	3602 (96.3)	712 (96.3)	2890 (96.3)
Yes	1355 (3.6)	24 (3.2)	111 (3.7)
Missing	4 (0.1)	3 (0.4)	1 (0.0)
Time since diagnosis			
5–10 years	1505 (40.2)	314 (42.5)	1191 (39.7)
11–15 years	1296 (34.6)	245 (33.2)	1051 (35.0)
16–20 years	940 (25.1)	180 (24.4)	760 (25.3)
BMI			
Underweight (< 18.5)	59 (1.6)	16 (2.2)	43 (1.4)
Normal weight (18.5–24.9)	2005 (53.6)	371 (50.2)	1634 (54.4)
Overweight (25.0 −29.9)	1217 (32.5)	242 (32.7)	975 (32.5)
Obesity (≥ 30.0)	459 (12.3)	110 (14.9)	349 (11.6)
Missing	1 (0.0)	-	1 (0.0)
Alcohol consumption			
Non-drinker = non-alcohol use	594 (15.9)	151 (20.4)	443 (14.8)
Former drinker	405 (10.8)	97 (13.1)	308 (10.3)
Drinker	2740 (73.2)	489 (66.2)	2251 (75.0)
Missing	2 (0.1)	2 (0.3)	-
Smoking status			
Non-smoker	2119 (56.6)	369 (49.9)	1750 (58.3)
Former smoker	1299 (34.7)	268 (36.3)	1031 (34.3)
Smoker	318 (8.5)	99 (13.4)	219 (7.3)
Missing	5 (0.1)	3 (0.4)	2 (0.1)
Substance use			
No	2808 (75.1)	546 (73.9)	2262 (75.3)
Yes	930 (24.9)	191 (25.8)	739 (24.6)
Missing	3 (0.1)	2 (0.3)	1 (0.0)
Physical activity, MVPA h/w, mean (SD)	13.0 (10.3)	14.0 (11.5)	12.7 (10.0)
Missing	44	14	30
Rheumatoid arthritis			
No	3614 (96.6)	692 (93.6)	2922 (97.3)
Yes	127 (3.4)	47 (6.4)	80 (2.7)
Diabetes mellitus			
No	791 (21.1)	204 (27.6)	587 (19.6)
Yes	97 (2.6)	26 (3.5)	71 (2.4)
Missing	2853 (76.3)	509 (68.9)	2344 (78.1)

*BMI* body mass index, *MVPA h/w* moderate and vigorous physical activity hours per week

^a^Together with partner, family or roommates

^b^AYAs could have received multiple treatments at primary cancer diagnosis

### Factors associated with self-reported peripheral neuropathic symptoms

Twelve factors, found to be associated with self-reported peripheral neuropathic symptoms (Table 2). Looking at the clinical factors, AYA survivors who were treated with chemotherapy, those with head and neck cancer, germ cell tumours, lymphoid haematological malignancies or thyroid cancer experienced self-reported peripheral neuropathic symptoms more frequently. AYA survivors who underwent stem cell therapy had lower odds of reporting peripheral neuropathic symptoms. Furthermore, AYA survivors who were more physically active, had rheumatoid arthritis and were former smokers or active smokers, had higher odds of self-reporting peripheral neuropathic symptoms. AYA survivors who drink alcohol or the diabetes status was unknown had lower odds of self-reporting peripheral neuropathic symptoms. Finally, looking at socio-demographic factors, AYA survivors who were older at the time of diagnosis, had primary education or none, secondary vocational educational, higher vocational education and were female more often reported peripheral neuropathic symptoms. AYA survivors with a partner were less likely to report peripheral neuropathic symptoms. The results of the multiple linear regression analysis, in which neuropathy was included as a continuous outcome variable, showed fairly similar statistically significant associated factors (Appendix 3—Table 2A). Differences were observed in physical activity and the tumour types head and neck cancer and lymphoid haematological malignancies, which were statistically significant in the multiple logistic regression, but not in the multiple linear regression analysis.

**Table 2:** Analysis of association of socio-demographic, clinical and health and lifestyle factors with self-reported peripheral neuropathic symptoms

		OR [95% CI]	*p*-value
Age at diagnosis		1.03 [1.01 – 1.05]	< 0.001*
Sex	Male	Reference	
	Female	1.37 [1.05 – 1.78]	0.019*
Partner	No	Reference	
	Yes	0.71 [0.54 – 0.95]	0.019*
Living situation	Alone	Reference	
	Together^1^	1.11 [0.80 – 1.54]	0.550
Social economic status	Low	Reference	
	Intermediate	0.99 [0.75 – 1.30]	0.915
	High	0.98 [0.76 – 1.28]	0.904
Level of education	Primary education, or none	2.84 [1.07 - 7.56]	0.036*
	Secondary education	1.21 [0.82 – 1.79]	0.331
	Secondary vocational education	1.30 [1.00 – 1.67]	0.049*
	Higher vocational education	1.40 [1.09 – 1.80]	0.008*
	University education	Reference	
Type of cancer	Bone and soft tissue sarcomas	1.66 [0.97 – 2.83]	0.065
	Breast	Reference	
	Central nervous system	1.39 [0.69 – 2.80]	0.362
	Colon and rectal	1.52 [0.78 – 2.96]	0.212
	Digestive tract, other	1.85 [0.68 – 5.02]	0.227
	Female genitalia	1.34 [0.88 – 2.02]	0.171
	Germ cell tumours (all sexes)	2.02 [1.30 – 3.14]	0.002*
	Head and neck	1.96 [1.06 – 3.64]	0.033*
	Lymphoid haematological malignancies	1.84 [1.03 – 3.28]	0.039*
	Male genitalia	0.00 [0.00 - . ]	0.999
	Melanoma	1.04 [0.60 – 1.81]	0.898
	Myeloid haematological malignancies	1.77 [0.80 – 3.89]	0.159
	Respiratory	2.52 [0.95 – 6.64]	0.063
	Thyroid	2.23 [1.36 – 3.65]	0.001*
	Urinary tract	0.76 [0.25 – 2.33]	0.632
	Other	1.54 [0.29 – 8.31]	0.615
Tumour stage	I	Reference	
	II	1.00 [0.78 – 1.28]	0.995
	III	1.09 [0.81 – 1.46]	0.580
	IV	1.07 [0.68 – 1.68]	0.769
	Unknown	1.37 [0.85 – 2.20]	0.195
Surgery^2^	No	Reference	
	Yes	1.03 [0.65 – 1.63]	0.916
Chemotherapy^2^	No	Reference	
	Yes	1.87 [1.43 – 2.46]	< 0.001*
Radiotherapy^2^	No	Reference	
	Yes	0.94 [0.77 – 1.16]	0.582
Hormonal therapy^2^	No	Reference	
	Yes	1.11 [0.78 – 1.58]	0.552
Targeted therapy^2^	No	Reference	
	Yes	0.73 [0.52 – 1.04]	0.081
Stem cell therapy^2^	No	Reference	
	Yes	0.43 [0.25 – 0.75]	0.003*
BMI	Underweight (< 18.5)	1.40 [0.76– 2.60]	0.284
	Normal weight (18.5 – 24.9)	Reference	
	Overweight (25.0 – 30.0)	1.03 [0.85 – 1.25]	0.740
	Obesity (> 30.0)	1.14 [0.88 – 1.49]	0.327
Time since diagnosis	5–10 years	Reference	
	11–15 years	0.86 [0.71 – 1.05]	0.136
	16–20 years	0.82 [0.66 – 1.03]	0.086
Alcohol consumption	Non-drinker = no alcohol use	Reference	
	Former drinker	0.90 [0.66 – 1.24]	0.530
	Drinker	0.70 [0.55 – 0.89]	0.003*
Smoking status	Non-smoker	Reference	
	Former smoker	1.28 [1.04 – 1.56]	0.018*
	Smoker	2.14 [1.58 – 2.88]	< 0.001*
Substance use	No	Reference	
	Yes	1.00 [0.81 – 1.25]	0.982
Physical activity (MVPA)		1.01 [1.00 – 1.05]	0.006*
Rheumatoid arthritis	No	Reference	
	Yes	2.08 [1.39 – 3.09]	< 0.001*
Diabetes mellitus	No	Reference	
	Yes	1.09 [0.65 – 1.83]	0.749
	Unknown	0.70 [0.56 – 0.87]	0.001*

X^2^ = 204.68 (*p*-value < 0.001) Nagelkerke R^2^ = 0.087. *p*-value < 0.05 is significant (bold *p*-values show a statistically significant OR). This multivariable model showed no multicollinearity

^1^Together with partner, family or roommates

^2^treatments were received at diagnosis. MVPA= moderate and vigorous physical activity. OR [95%CI] = odds ratio and 95% confidence interval

## Discussion

This study investigated the prevalence of and factors associated with self-reported peripheral neuropathic symptoms in long-term AYA cancer survivors in the Netherlands. The results of this study showed that self-reported peripheral neuropathic symptoms persist in 1 out of 5 participating AYA cancer survivors up to 5–20 years after diagnosis. In addition, self-reported peripheral neuropathic symptoms are associated with multiple clinical factors (including type of cancer, chemotherapy and stem cell therapy), health and lifestyle factors (including alcohol consumption, smoking status, physical activity, and rheumatoid arthritis) and socio-demographic factors (including age at diagnosis, sex, level of education and partner status).

Due to the lack of AYA-specific research focusing on self-reported peripheral neuropathic symptoms, it is not possible to compare the prevalence of our study with other studies. Looking at research focusing on adult cancer survivors, we see that the prevalence in our study with AYAs is lower than in studies with adult cancer survivors. A systematic review by Seretny et al. [15], showed that 30% of adult cancer survivors experienced neuropathy 6 months or later after treatment. A possible explanation for the lower prevalence is the difference in age between the two studies as several studies have shown that older patients have a higher risk of neuropathy [15, 23]. This is supported by our results, in which AYAs with an older age at diagnosis were more likely to report peripheral neuropathic symptoms than younger AYAs. A possible explanation for this phenomenon is that the number of comorbidities, such as diabetes mellites, increases with age. In addition, the regenerative capacity of the peripheral nervous system decreases over time, which may contribute to a higher prevalence of neuropathy symptoms [31]. Furthermore, most of the articles included within the systematic review by Seretny et al. [15] included patient groups that were also more likely to report neuropathy within our study, such as colorectal and breast cancer. Moreover, the study by Seretny et al. [15] focused specifically on patients with CIPN and our study does not solely focus on patients treated with chemotherapy, while chemotherapy emerges as a risk factor for neuropathy [32–34]. Finally, our study only included patients who were at least 5 years post-treatment, whereas Seretny et al. [15] assessed prevalence at 6 months and longer post-treatment. As neuropathy symptoms typically appear during treatment and gradually decrease and stabilise over time, this difference in follow-up time may explain the difference in prevalence between studies [23].

Based on our results, the prevalence of self-reported peripheral neuropathic symptoms in the AYA study population is influenced by an interplay of several factors. Our study showed that patients treated with chemotherapy were more likely to experience peripheral neuropathic symptoms than patients who did not receive chemotherapy, which is in line with previous research [32–34]. This can be explained by the fact that chemotherapeutic agents can damage the structures of the nervous system which can lead to CIPN [33]. The prevalence of CIPN is most common with chemotherapies based on platinum-based drugs, taxanes and vinca alkaloids, which is commonly used in lung cancer, breast cancer and ovarian cancer, for example [32, 33, 35]. In addition, the severity of neuropathic symptoms is proportional to the cumulative dose of the drug [32, 33, 35]. Despite the fact that this cannot be concluded based on our study results, due to the lack of information on the type and dose of chemotherapy the prevalence of neuropathy does seem to be higher in the groups generally known to be treated more often with these types of chemotherapy. However, the numbers of included patients with these tumour types should also be taken into account here.

Also, several socio-demographic factors were associated with peripheral neuropathic symptoms, including age at diagnosis and sex. As discussed previously, age affects the rate of comorbidities and the regenerative capacity of the peripheral nervous system [31]. For sex, our study showed that women were more likely to report peripheral neuropathic symptoms than men. This is in line with previous scientific research, showing that women experience more toxicity than men [36, 37]. Men used to be mainly represented in clinical trials and therefore the dosage of, for example, chemotherapy, is often based on the male body [36]. As a result, due to differences in body composition, women receive relatively higher dosages of chemotherapy than men. Following this, it may explain why women experience more neuropathy symptoms as the dose of the chemotherapy is proportional to the severity of symptoms [32–34]. Another explanation is that women and men react differently to drugs because of biological differences between the sexes [38]. For example, research showed that men break down drugs faster than women, resulting in lower drug levels [36, 38]. In addition, men and women have different types of tumours, which require a different treatment. It could be that women are more often treated with agents that cause more neuropathy symptoms than men, e.g. weekly paclitaxel in breast cancer. Relatively higher doses of chemotherapy, slower drug degradation and differences in tumour types may explain why women are more likely to experience neuropathy than men.

In addition, our study shows that AYAs who have a partner were less likely to self-report peripheral neuropathic symptoms than AYAs without a partner. A possible explanation could be that a partner can encourage adopting healthier life habits or seeking medical help at the first sign of symptoms. A partner can provide emotional and practical support, which may lead to patients experiencing symptoms less intense. This may eventually lead to less reporting of symptoms in AYAs who have a partner.

Finally, several health and lifestyle factors also play a role in the development of peripheral neuropathic symptoms. Our results showed that smokers and former smokers were more likely to experience self-reported peripheral neuropathic symptoms than non-smokers. This can be explained by the fact that smoking affects the nerves’ oxygen supply. Nicotine constricts arteries, reducing blood flow, especially in the extremities [39]. At the same time, tobacco smoke contains carbon monoxide, which binds to haemoglobin faster than oxygen, displacing oxygen from the blood [39]. This can make the nerves in the extremities less oxygenated, which negatively affects their functionality, and therefore might cause peripheral neuropathic symptoms [40].

Unlike smoking, the results in this study suggest that alcohol consumption is associated with a lower risk of self-reporting peripheral neuropathic symptoms, while research has shown that prolonged and excessive alcohol consumption can lead to neuropathy [17, 41]. A possible explanation for the difference could be that drinking alcohol in our study was defined as at least one glass of alcohol per week, while self-reported peripheral neuropathic symptoms only occur as a consequence of higher intakes of alcohol per week. It is likely that the clinically relevant effect—caused by chronic excessive alcohol consumption—was not identified due to the broad nature of this category. Future research with more differentiation between the level of alcohol consumption per week could possibly explain the difference between the results of our research and other studies.

Also, our results showed that AYAs who were more physically active had a higher odds of reporting neuropathic symptoms. This contradicts previous research which shows that physical activity can play a protective role in the development of neuropathic symptoms [16, 42]. A possible explanation for this difference is that the level of physical activity in our study population was relatively high, with an average of 13.0 h of moderate to vigorous physical activity per week. This may mean that the analyses did not sufficiently reflect the difference between low and high levels of physical activity.

Similarly, having diabetes mellitus does not emerge as a factor increasing the likelihood of reporting symptoms of peripheral neuropathy, even though it is known to be a risk factor for peripheral neuropathy symptoms [17]. A possible explanation for this result is that the prevalence of diabetes mellitus within our study population is relatively low. Furthermore, for a large group of participants, it is unknown whether they have diabetes mellitus or not, which may have significantly impacted our results. The absence of a significant association in our study is therefore more likely to reflect a limitation in data completeness than a true lack of effect. Our results should therefore be interpreted with caution.

The role of BMI in the development of peripheral neuropathic symptoms is less clear. In the current literature, there is no consensus on whether BMI is associated with neuropathy [23, 43]. In this study, we found no association between BMI and self-reported peripheral neuropathic symptoms. A possible explanation for these different results between studies is how BMI is treated as a variable in the analyses among studies (with or without the underweight category, and/or overweight and obesity as separate or joint categories). Another possible explanation is that BMI may not be the best way to measure overweight and obesity. BMI does not represent a patient’s complete health status because it does not account for differences in body composition [44]. Other options, such as body adiposity index, may be more accurate, but are less practical to use [44]. To accurately determine this relationship, we need to identify and measure an appropriate, uniform outcome using an appropriate tool, as well as analyse it the same way as well. Lastly, it should be noted that all BMI-related information refers to the moment of study participation: participants’ BMI at cancer diagnosis was unknown.

The strengths and uniqueness of this cohort study are its very large sample size, long-term follow-up of more than five years, population-level data and inclusion of a wide range of cancer types. However, this study also has some limitations that should be mentioned. Firstly, the NCR only includes primary treatment, but excluding follow-up treatments, which may lead to an under- or overestimation of peripheral neuropathic symptoms associations. In line with this, no information regarding treatment regimen or dose was available. Second, because of the cross-sectional design of the study, risk factors cannot be directly examined, but only associated factors. Third, the questionnaire used in the SURVAYA study makes it only possible to determine the prevalence of peripheral neuropathic symptoms and not the severity thereof. In addition, the questionnaire only measures tingling and numbness symptoms and not, for example, pain symptoms while these can also occur in neuropathy. Also, in this manuscript, neuropathic symptoms were self-reported and not clinically verified. Taking altogether, the combination of health professional-based assessment tools and PROMs is recommended to best evaluate the patients’ CIPN-related symptoms. For future studies on peripheral neuropathic symptoms in AYAs or in identified risk groups in AYAs, it may be interesting to use questionnaires that assess both the presence of multiple neuropathic symptoms (tingling, numbness and pain) and the severity of neuropathic symptoms to determine the impact on daily life [45, 46]. In line with this, some AYAs seemed to experience peripheral neuropathic symptoms while they were not treated with chemotherapy. This may partly be due to the way peripheral neuropathic symptoms were measured in this study, using two items of a PROM, potentially leading to high(er) levels of peripheral neuropathic symptoms while it more so represents other symptoms, such as sensory neuropathy (such as hearing problems), decreased muscle strength, or tripping due to problems with sight. The tingling and numbness symptoms could also be a symptom of other comorbidities, such as rheumatoid arthritis, instead of solely neuropathy, potentially introducing bias. A two-item scale was used to assess neuropathic symptoms. Such short scales may limit reliability and content validity; however, the two items used in this study directly capture core patient-reported manifestations of peripheral neuropathic symptoms, which are widely accepted as key symptoms in oncology survivorship research. Moreover, short symptom scales are common in EORTC modules, where the focus is on clinical relevance and feasibility rather than scale length. For the transformation of the neuropathic symptoms score to a 0–100 score, the standardized EORTC scoring procedures were followed to ensure comparability with other studies. These linear transformations are well-established and do not alter the underlying distributional properties of the data. Although dichotomising continuous variables can lead to loss of information and statistical power, this step was applied to facilitate clinical interpretability by identifying a subgroup with relatively elevated symptom burden. We performed a sensitivity analysis (multiple linear regression analysis) in which neuropathy was analysed as a continuous outcome variable. The results of this analysis were largely in line with those of the multiple logistic regression analysis. This suggests that dichotomising the outcome measure did not substantially influence the results of our study. The threshold of ≥ 10 points above the sample mean was chosen to reflect a meaningful deviation from the average symptom level, consistent with prior work using EORTC scales. Finally, the SURVAYA study questionnaire consists of 40 pages. This could have led to healthy survivorship bias, because AYAs experiencing problems may not have participated in the study or completed the questionnaire, which could lead to an underestimation of the prevalence of peripheral neuropathic symptoms [47]. However, the reasoning could also be the other way around, that AYA survivors who experience symptoms want to be heard and therefore participated in the study. The generalizability of the results is also likely affected by the SURVAYA study recruitment rate of 36%. Therefore, the findings described in this manuscript should be interpreted as descriptive associations rather than generalizable prevalence estimates.

### Implications for clinical practice and future research

Compared with older adults, AYAs have a relatively long life expectancy and thus a risk of long-term effects. It is crucial to consider socio-demographic characteristics, as well as lifestyle choices and clinical factors to develop effective strategies for reducing and managing peripheral neuropathic symptoms. The results of this study highlight the high prevalence of, and associated factors with, peripheral neuropathic symptoms in the participating long-term AYA cancer survivors. These results can be used by healthcare providers to further identify high risk groups within the AYA cancer survivor population who might need additional monitoring. In order to reliably understand the course and impact of peripheral neuropathic symptoms, future research should use a validated scoring method. This should start with baseline scores, which are then followed up several times over time. This will enable the longitudinal determination of changes in symptoms and level of function, facilitating the more precise mapping of risk groups among AYA cancer survivors. These studies should use standardized, validated questionnaires for self-reported peripheral neuropathic symptoms, as well as record the tumour type and type and dosage of treatment patients received. This should preferably be done in a large-scale prospective longitudinal study.

## Conclusion

To conclude, in this Dutch study, self-reported peripheral neuropathic symptoms were associated with chemotherapy exposure in long-term AYA cancer survivors. In addition, self-reported peripheral neuropathic symptoms were associated with other clinical factors (including type of cancer and stem cell therapy), health and lifestyle factors (including alcohol consumption, smoking status, physical activity, and rheumatoid arthritis) as well as sociodemographic factors (including age at diagnosis, sex, level of education and partner status). Future longitudinal studies including baseline assessments are needed to better understand the development and course of neuropathy in this population.

## Supplementary Information

Below is the link to the electronic supplementary material.

ESM 1 (DOCX 44.2 KB)

## Data Availability

The dataset generated and analyzed in this study is available from the corresponding author on reasonable request. The data are not publicly available due to privacy issues.
