# Supplementary material for: Self-reported peripheral neuropathic symptoms in long-term adolescent and young adult (AYA) cancer survivors: results of the SURVAYA study
Source: Support Care Cancer. 2026 Jul 2;34(7):719. doi: 10.1007/s00520-026-10912-7 (PMC13328226; doi:10.1007/s00520-026-10912-7)
Supplement: Supplementary file 1 — (DOCX 44.2 KB) [file 520_2026_10912_MOESM1_ESM.docx]

**APPENDIX 1 – Flowchart inclusion process**

**Inclusion AYA cancer survivors in the study**

**Population-based study population selected from the Netherlands Cancer Registry (NCR) (n=17,098):**

- AYA cancer survivors diagnosed in 1999-2000-2014/2015 at age 18-39 years
- Treated in an Academic hospital or Netherlands Cancer Institute
- All malignant tumour types of all stages
- Alive at time of invitation, which started in May 2019 and ended in June 2021

**Excluded**

- Excluded tumour types, for multifactorial reasons, mainly based on very good prognosis or extreme rarity (n=962)
- No permission of hospitals (n=3192)
- Not able to link with municipal personal records database to obtain up-to-date addresses and vital status (n=1053)

**Included (N=11,891)**

- Eligible AYA cancer survivors who were invited to participate in the study

**Excluded**

- Non-verified addresses (n=515)
- Not able to participate (participation in other study, language problems or cognitive problems or indicated that they did not have cancer (n=80)

**Included (N=11,296)**

- AYA cancer survivors included for analysis

**Respondents (N=4010)**

- AYA cancer survivors included

**Non-respondents (N=7286)**

**Excluded (N=269)**

- AYA cancer survivors without data about peripheral neuropathic symptoms

**Included (N=3741)**

- AYA cancer survivors with data about peripheral neuropathic symptoms

Figure A1: Flowchart of study participants in the study (black inclusion in the SURVAYA study and green inclusion current analyses)

**APPENDIX 2 - Table A1**

**Table A1:** Differences in characteristics of the included and excluded AYA survivors

|  | **Included AYA survivors** | **Excluded AYA survivors** | **p-value** |
| --- | --- | --- | --- |
|  | **N=3741** | **N=269** |  |
|  | N (%) | N (%) |  |
| **Sex**  Male  Female | 1456 (38.9)  2285 (61.1) | 93 (34.6)  176 (65.4) | 0.157 |
| **Age at diagnosis, in years, mean(SD)** | 31.6 (5.9) | 31.5 (5.4) | 0.874 |
| **Partner status**  Partner  Single  Missing | 3109 (83.1)  619 (16.5)  13 (0.3) | 224 (83.3)  42 (15.6)  3 (1.1) | 0.730 |
| **Living situation**  Together^1^  Alone | 3282 (87.7)  459 (12.3) | 235 (87.4)  34 (12.6) | 0.858 |
| **Socials economic status**  Low  Intermediate  High  Missing | 499 (13.3)  1151 (30.8)  2077 (55.5)  14 (0.4) | 45 (16.7)  84 (31.2)  140 (52.0)  - | 0.262 |
| **Educational level**  Primary education, or none  Secondary education  Secondary education vocational  Higher vocational education  University education  Missing | 24 (0.6)  250 (6.7)  1354 (36.2)  1276 (34.1)  829 (22.2)  8 (0.2) | 4 (1.5)  16 (5.9)  102 (37.9)  98 (36.4)  49 (18.2)  - | 0.273 |
| **Type of cancer** |  |  | 0.238 |
| Bone and soft tissue sarcomas | 165 (4.4) | 7 (2.6) |  |
| Breast | 885 (23.7) | 59 (21.9) |  |
| Central nervous system | 142 (3.8) | 8 (3.0) |  |
| Colon and rectal | 76 (2.0) | 6 (2.2) |  |
| Digestive tract, other | 30 (0.8) | 1 (0.4) |  |
| Female genitalia | 407 (10.9) | 38 (14.1) |  |
| Germ cell tumours | 652 (17.4) | 40 (14.9) |  |
| Head and neck | 116 (3.1) | 8 (3.0) |  |
| Lymphoid haematological malignancies | 555 (14.8) | 36 (13.4) |  |
| Male genitalia | 5 (0.1) | 1 (0.4) |  |
| Melanoma | 262 (7.0) | 28 (10.4) |  |
| Myeloid haematological malignancies | 140 (3.7) | 8 (3.0) |  |
| Respiratory | 27 (0.7) | 3 (1.1) |  |
| Thyroid gland | 230 (6.1) | 18 (6.7) |  |
| Urinary tract | 39 (1.0) | 7 (2.6) |  |
| Other | 10 (0.3) | 1 (0.4) |  |
| **Tumour stage**  I  II  III  IV  Unknown | 1600 (42.8)  990 (26.5)  536 (14.3)  172 (4.6)  443 (11.8) | 126 (46.8)  73 (27.1)  37 (13.8)  7 (2.6)  26 (9.7) | 0.376 |
| **Surgery^2^**  No  Yes  Missing | 818 (21.9)  2919 (78.0)  4 (0.1) | 62 (23.0)  207 (77.0)  - | 0.657 |
| **Chemotherapy^2^**  No  Yes  Missing | 1633 (43.7)  2104 (56.2)  4 (0.1) | 134 (49.8)  135 (50.2)  - | 0.051 |
| **Radiotherapy^2^**  No  Yes  Missing | 1959 (52.4)  1778 (47.5)  4 (0.1) | 145 (53.9)  124 (46.1)  - | 0.638 |
| **Hormone therapy^2^**  No  Yes  Missing | 3282 (87.8)  455 (12.2)  4 (0.1) | 240 (89.2)  29 (10.8)  - | 0.498 |
| **Targeted therapy^2^**  No  Yes  Missing | 3447 (92.1)  290 (7.8)  4 (0.1) | 251 (93.3)  18 (6.7)  - | 0.525 |
| **Stem cell therapy^2^**  No  Yes  Missing | 3602 (96.3)  135 (3.6)  4 (0.1) | 262 (97.4)  7 (2.6)  - | 0.387 |
| **Time since diagnosis**  5-10 years  11-15 years  16-20 years | 1505 (40.2)  1296 (34.6)  940 (25.1) | 122 (45.4)  89 (33.1)  58 (21.6) | 0.214 |
| **BMI**  Underweight (<18.5)  Normal weight (18.5 – 24.9)  Overweight (25.0 – 29.9)  Obesity (≥30.0)  Missing | 59 (1.6)  2005 (53.6)  1217 (32.5)  459 (12.3)  1 (0.0) | 3 (1.1)  114 (42.4)  62 (23.0)  90 (33.5)  - | **<0.001*** |
| **Alcohol consumption**  Non-drinker = non-alcohol use  Former drinker  Drinker  Missing | 594 (15.9)  405 (10.8)  2740 (73.2)  2 (0.1) | 47 (17.5)  25 (9.3)  173 (64.3)  24 (8.9) | 0.394 |
| **Smoking status**  Non-smoker  Former smoker  Smoker  Missing | 2119 (56.6)  1299 (34.7)  318 (8.5)  5 (0.1) | 136 (50.6)  89 (33.1)  29 (10.8)  15 (5.6) | 0.254 |
| **Substance use**  No  Yes  Missing | 2808 (75.1)  930 (24.9)  3 (0.1) | 169 (62.8)  59 (21.9)  41 (15.2) | 0.735 |
| **Physical activity,** **MVPA hours per week, mean(SD)**  Missing | 13.0 (10.6)  44 | 13.6 (12.3)  200 | 0.707 |
| **Rheumatoid arthritis**  No  Yes  Missing | 3614 (96.6)  127 (3.4)  - | 21 (7.8)  -  248 (92.2) | 0.390 |
| **Diabetes myelitis**  No  Yes  Missing | 791 (21.1)  97 (2.6)  2853 (76.3) | 7 (2.6)  1 (0.4)  261 (97.0) | 0.887 |

^1^ together with partner, family or roommates; ^2^ the treatments were received at primary diagnosis; BMI= Body mass index; MVPA= moderate and vigorous physical activity, * statistically significant

**Appendix 3 - Table A2**

**Table A2:** Analyses of association of socio-demographic, clinical and health and lifestyle factors with self-reported peripheral neuropathic symptoms (multiple linear regression analyses)

|  | | B [95% CI] | p-value |
| --- | --- | --- | --- |
| Age at diagnosis | | 0.25 [0.11 – 0.38] | **<0.001*** |
| Sex | Male  Female | Reference  3.50 [1.34 – 5.66] | **0.001*** |
| Partner | No  Yes | Reference  -3.08 [-5.60 – -0.57] | **0.016*** |
| Living situation | Alone  Together^1^ | Reference  1.61 [-1.24 – 4.45] | 0.268 |
| Social economic status | Low  Intermediate  High | Reference  -0.53 [-2.88 – 1.82]  -1.32 [-3.52– 0.90] | 0.657  0.243 |
| Level of education | Primary education, or none  Secondary education  Secondary vocational education  Higher vocational education  University education | 15.64 [5.86 – 25.43]  -0.17 [-3.39 – 3.05]  2.54 [0.53 – 4.55]  2.97 [1.02 – 4.91]  Reference | **0.002***  0.918  **0.013***  **0.003*** |
| Type of cancer | Bone and soft tissue sarcomas | 4.47 [0.15 – 8.79] | 0.065 |
|  | Breast | Reference |  |
|  | Central nervous system | 5.04 [ -0.98 – 11.06] | 0.100 |
|  | Colon and rectal | 4.17 [ -1.50 – 9.83] | 0.149 |
|  | Digestive tract, other | 6.19 [-2.39 – 14.77] | 0.157 |
|  | Female genitalia | 0.97 [ -2.43 – 4.36] | 0.577 |
|  | Germ cell tumours (all sexes) | 6.71 [3.09 – 10.33] | **<0.001*** |
|  | Head and neck | 4.84 [-0.26 – 9.92] | 0.062 |
|  | Lymphoid haematological malignancies | 5.02 [-0.02 – 10.06] | 0.051 |
|  | Male genitalia | -2.57 [-22.02 – 16.87] | 0.796 |
|  | Melanoma | 0.49 [-3.55 – 4. 53] | 0.812 |
|  | Myeloid haematological malignancies | 6.86 [ -0.04 – 13.77] | 0.051 |
|  | Respiratory | 8.26 [-0.51 – 17.03] | 0.065 |
|  | Thyroid | 6.19 [2.01 – 10.38] | **0.004*** |
|  | Urinary tract | -1.68 [-9.19 – 5.83] | 0.660 |
|  | Other | 0.99 [-13.07 – 15.04] | 0.891 |
| Tumour stage | I  II  III  IV  Unknown | Reference  0.45 [-1.59 – 2.48]  2.27 [-0.23 – 4.77]  1.07 [-2.81 – 4.94]  1.58 [-2.55 – 5.70] | 0.668  0.076  0.590  0.453 |
| Surgery^2^ | No  Yes | Reference  -0.10 [-4.23 – 4.04] | 0.963 |
| Chemotherapy^2^ | No  Yes | Reference  5.12 [2.93 – 7.32] | **<0.001*** |
| Radiotherapy^2^ | No  Yes | Reference  -0.42 [-2.14 – 1.30] | 0.633 |
| Hormonal therapy^2^ | No  Yes | Reference  -0.52 [-3.44 – 2.39] | 0.725 |
| Targeted therapy^2^ | No  Yes | Reference  -2.86 [-5.74 – 0.03] | 0.052 |
| Stem cell therapy^2^ | No  Yes | Reference  -5.38 [-9.83 – -0.93] | **0.018*** |
| BMI | Underweight (<18.5)  Normal weight (18.5 – 24.9)  Overweight (25.0 – 30.0)  Obesity (>30.0) | 1.60 [-4.13 – 7.33]  Reference  0.07 [-1.54 – 1.68]  2.03 [-0.30 – 4.36] | 0.584  0.933  0.088 |
| Time since diagnosis | 5-10 years  11-15 years  16-20 years | Reference  -0.52 [-2.17 – 1.14]  -0.60 [-2.47 – 1.27] | 0.543  0.531 |
| Alcohol consumption | Non-drinker = no alcohol use  Former drinker  Drinker | Reference  -0.45 [-3.31 – 2.42]  -3.13 [-5.25 – -1.02] | 0.760  **0.004*** |
| Smoking status | Non-smoker  Former smoker  Smoker | Reference  2.36 [0.69 – 4.03]  5.85 [3.08 – 8.63] | **0.006***  **<0.001*** |
| Substance use | No  Yes | Reference  0.44 [-1.37 – 2.26] | 0.632 |
| Physical activity (MVPA) | | 0.05 [-0.02 – 0.12] | 0.177 |
| Rheumatoid arthritis | No  Yes | Reference  7.94 [3.99 – 11.88] | **<0.001*** |
| Diabetes mellitus | No  Yes  Unknown | Reference  1.60 [-3.91 – 6.40]  -3.76 [-5.72 – -1.81] | 0.512  **0.001*** |

F=5.00 (p-value <0.001) Adjusted R^2^ = 0.051. p-value <0.05 is significant (bold p-values show a statistically significant B). This multivariable model showed no multicollinearity. ^1^Together with partner, family or roommates.
^2^ treatments were received at diagnosis. MVPA= moderate and vigorous physical activity. [95%CI] = 95% confidence interval
